# The Observation of the Change of TCE Caused by Different Acupuncture Stimulation

**DOI:** 10.1155/2013/856905

**Published:** 2013-08-29

**Authors:** Tao Huang, Xinnong Cheng

**Affiliations:** Institute of Acupuncture and Moxibustion, China Academy of Chinese Medical Science, No. 16 Nanxiaojie of Dongzhimennei, Beijing 100700, China

## Abstract

*Purpose*. To observe the change of transcutaneous CO_2_ emission on meridian points or nonacupoints when the different needle sensations were gotten and study the associativity between Deqi acupuncture and periphery constitution energy metabolism effect. *Method*. 20 healthy volunteers were punctured on Neiguan (P6) in different ways including sham, shallow, Deqi acupuncture, and Deqi plus pressed P5, and measured TCE of different points before, during, and after acupuncture. *Result*. Needle sensations of sham acupuncture and shallow acupuncture were less than those of Deqi acupuncture. TCE of meridian points increased significantly and showed the specificity of meridian/channels. *Conclusion*. Verum acupuncture could cause the stronger needling sensations including distention, aching, numbness, and tingling than sham and shallow acupuncture. The strength of needling sensation caused by Deqi acupuncture is moderate and brought the best curative effects in TCE measurement. Deqi acupuncture could improve the energy metabolism of the points on the corresponding meridian/channel.

## 1. Introduction

Usually, effective acupuncture is also called Deqi acupuncture (arrival of Qi) [1]. Deqi/Qi arrival (with its uniquely human characteristics like Qigong and Yin-Yang) is accepted by parts of the international academic community [2]. When inserting the needle to a certain depth, both the acupuncturist and the patient will feel something is changing; this means Qi arrival or Deqi sensation. Deqi is an important part of acupuncture or meridian/channel researches, a main way of judgment of acupuncture effects and clinical curative effects [3].

The experiment design of double blind, control, and random is the golden standard in the Western medicine scope. In many acupuncture clinical trials, including the famous reports from Germany study groups, the acupuncture curative effects are queried because of the placebo or sham acupuncture design [4].

So, it is a challenge that we must face: whether the efficiency of acupuncture is better than placebo or, further, the acupuncture is only playing a role of psychology comfort.

To answer this question, therefore, we did a group of experiments for the comparison between placebo acupuncture, sham acupuncture, and verum acupuncture. 

We did a serious transcutaneous CO_2_ emission measurement on acupoints using Fewil Q.F. microdetector of CO_2_ emission (which originally was invented by Professor W. Franyo, remade by Professor Zhang WB, Beijing, China) to study the curative effects of acupuncture. The previous experiments showed that the transcutaneous carbon dioxide (CO_2_) emission (TCE) could reflect the skin tissue energy metabolism to a certain degree. And TCE will be a sensitive index to observe the acupuncture curative effects [4].

According to Professor Hu's studies, pressure could block the acupuncture effects [5]. We specially designed that using 1 kg pressure on the channel of Pericardium, and observed the change of needling sensation and TCE of the point.

## 2. Methods and Material

### 2.1. Selection of Study Participants

Through advertisements on the campuses, 20 healthy volunteers (8 male, 12 female) with a mean age of 29.00 ± 9.22 years were enrolled among the staffs and graduate students from the Institute of Acupuncture and Moxibustion of the China Academy of Chinese Medical Sciences. All participants who had received acupuncture before, could distinguish the needle sensation and gave informed consent. The experimental procedure was approved by the Ethics Committee of the Institute of Acupuncture and Moxibustion of China Academy of Chinese Medical Sciences.

### 2.2. Acupuncture

Each volunteer underwent 4 measurements (two different kinds of sham acupuncture and two different kinds of verum acupuncture, see below) in random order every other day. To avoid discrepancies in manipulation, all acupuncture operations were performed by the same medical practitioner. 

**Figure 1:**
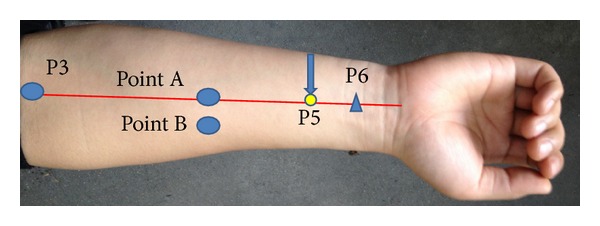
The location of the points.

The volunteers lay down on the back and exposed the right arm, so the acupoint Neiguan (P6), Quze (P3), and other observational points could be marked in accordance with a textbook on acupuncture and moxibustion [1] (see Figure 1). Needle retaining time was about 20 minutes.

### 2.3. Acupuncture Point

Neiguan (P6) is located on the pericardium meridian, 2 cun below wrist crease, between 2 tendons. All the locations of acupoint including the following were mentioned in Professor Cheng'* Chinese Acupuncture and Moxibustion* [6].

### 2.4. Measurement Points

Quze (P3), is located on the pericardium meridian, in the elbow fossa, on the elbow crease, ulnar of biceps brachii tendon.

 Point A: located on the pericardium meridian, in the middle between P3 and P5.
 Point B: 1 cm besides Point A, radial side.

### 2.5. Pressed Point

Jianshi (P5): 1 cun below P6.

### 2.6. Sham Acupuncture

#### 2.6.1. Placebo

**Figure 2:**
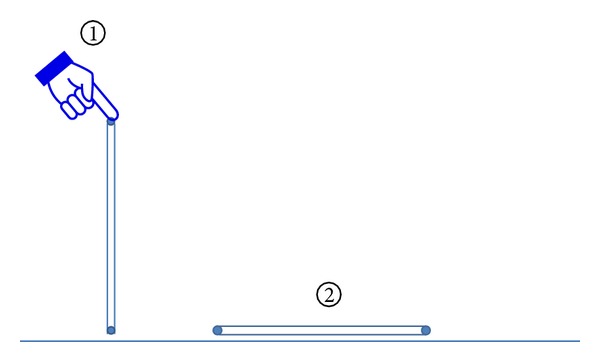
Sham acupuncture.

Sham acupuncture was performed using a single-use acupuncture needle tube (Tianxie brand, Suzhou, China) which was tapped on P6, but no needle was inserted (see Figure 2).

### 2.7. Shallow Acupuncture

**Figure 3:**
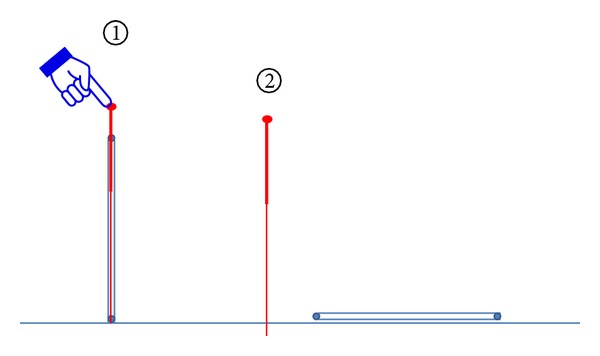
Shallow acupuncture.

Shallow acupuncture was performed using a single-use acupuncture needle with tube (Tianxie brand, Suzhou, China) which was tapped on P6; the insertion of needle into the skin was only 1 or 2 mm. (see Figure 3). 

### 2.8. Verum Acupuncture

#### 2.8.1. Deqi Acupuncture

**Figure 4:**
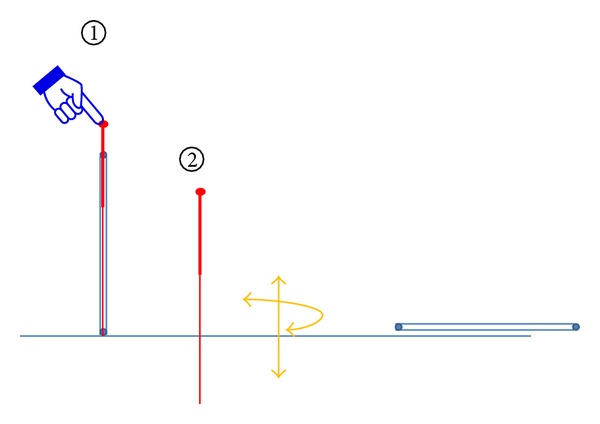
Verum acupuncture.

Acupuncture stimulation was done manually, using single-use acupuncture needle (0.25 × 25 mm, Tianxie brand, Suzhou, China). The doctor inserted the needle on P6 through a tube to retain depth and then repeated lifting, thrusting, twisting, and rotating until both the practitioner and the volunteer felt the Qi arrival or needling sensations like soreness, numbness, aching, pressure, or tingling; then the insertion was stopped. The needle was remained in place for 20 mins and then removed (see Figure 4).

#### 2.8.2. Acupuncture and Pressure on P5

**Figure 5:**
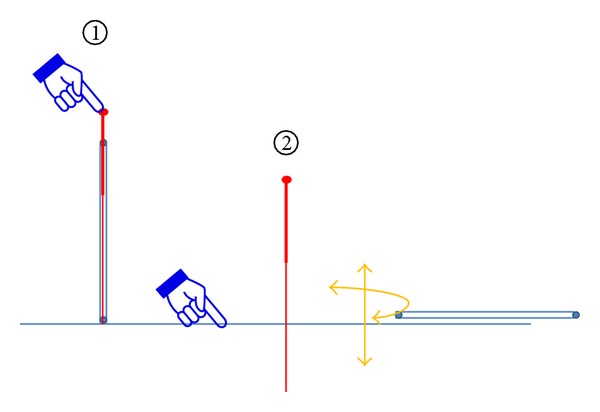
Acupuncture and pressed P5.

A similar operation like pure acupuncture after the needle insertion was performed until the Qi arrival. Then, manipulator used a spring manometer which fixed in a universal bracket pressed on P5 with 9.8 N pressure (Pressure area 1 × 1 cm). The pressure was controlled by adjusting the height of the gauge. The needle was left in P6 for 20 mins before its removal (see Figure 5).

### 2.9. Measurement

#### 2.9.1. TCE Measurements

**Figure 6:**
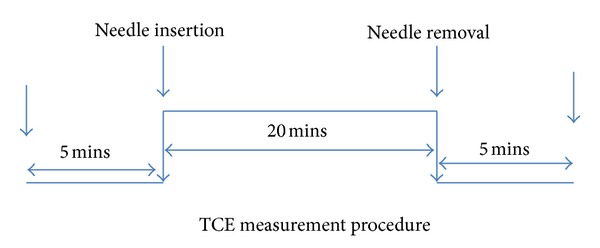
TCE measurement procedure.

The Fewil Q. F. carbon dioxide measuring instrument was designed by Professor W. Franyo (Hungary) in 1976 and improved by Professor Zhang WB (China) in 1996, which could measure the micro-carbon dioxide of the skin (see Figure 6).

Before the experiments, the fan and the air-conditioner were turned on, so the lab was at well ventilated and constant temperature (26 ± 1°C) and humidity (40–60%). The laboratory technician and the doctor wore tasks to avoid influencing the accuracy of the instrument.

**Figure 7:**
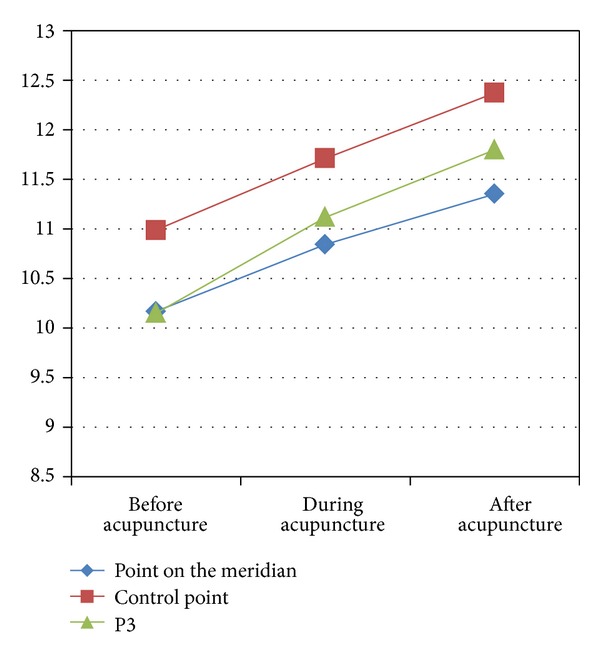
The change of point's TCE caused by sham acupuncture.

The measurement procedure was like below (see Figure 7).

Firstly, the instrument measured the air carbon dioxide of the room 3 times. And then, the technician measured the transcutaneous carbon dioxide (CO_2_) emission of P3, Point A and B on the skin 3 times before, during, and after sham or verum acupuncture.

#### 2.9.2. The Inquiry and Record of Needling Sensation

During the experiment, all volunteers wore eyeshade and were asked the feeling using for reference the needling sensations questionnaire which is made by Harvard Medical School [6] and, respectively, assessed by visual analogue scale (VAS). Zero means “no sensation at all,” and 10 means “too much to bear.” 

The questions were unified:

  “I will puncture you. Do you have something special?
 Do you feel aching, soreness … and so on?
 If yes, please describe it and tell us the degree. 0 means nothing and 10 stands for too much to bear.”

## 3. Analysis of Data

The average of 3 times measurement of CO_2_ of the points and the room air was calculated. M stood for the TCE of points and N for the air. Considering the influence of air, the modified TCE = *M* − (*N*∗0.1). One percent is coefficient based on past experience. 

A one-tailed Fisher's exact test was used to analyze a possible connection between stimulation and perception. A *P* value of <0.05 was considered significant.

## 4. Results

### 4.1. The Needling Sensation on Acupuncturing P6

Recording and comparing 20 volunteers' needling sensations, it was found that the most commonly appeared sensations were distention and heaviness (pressure), the other were aching, numbness, tingling, and hotness in the same order of occurance (see Table 1).

The needling sensation degree was different according to the stimulation way. It was interesting that even though the acupuncturist did give verum needling for the volunteers, some of them still felt distention or numbness feeling. But the feeling was very mild and did not exceed 3 in VAS.

For the Deqi acupuncture, all the volunteers could feel needling sensations including heaviness, distention, aching, numbness, tingling, and hotness. The strength of sensations was between 2 and 6 and was not very strong, but that of the stimulation caused by Deqi acupuncture plus pressed P5 was much stronger than pure acupuncture. The VAS was between 4 and 9 (see Table 1).

In the group of sham acupuncture, the change tendency of TCE of Points A (on the meridian), B (control point), and P3 (Quze) was almost the same and without significance. The TCE of all the points increased over the time.

But in the other 3 groups, despite the shallow acupuncture, the TCE of P3 and Point A increased during acupuncture with significance, while the TCE of Point B decreased. Among these acupuncture experiments, the change of TCE of the Deqi acupuncture which increased significantly was the biggest. 

When we did sham acupuncture, TCE of all the points was increased almost evenly (see Figure 7).

**Figure 8:**
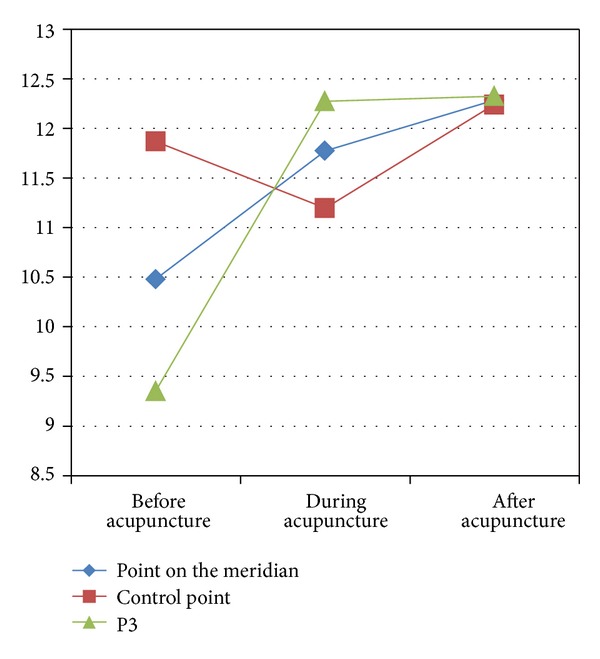
The change of points' TCE caused by shallow acupuncture.

But when the skin got stimulated, even though very mildly like shallow acupuncture, TCE of the point on the meridian and P3 was increased in different way while that of the control point decreased first and then increased (see Figure 8).

**Figure 9:**
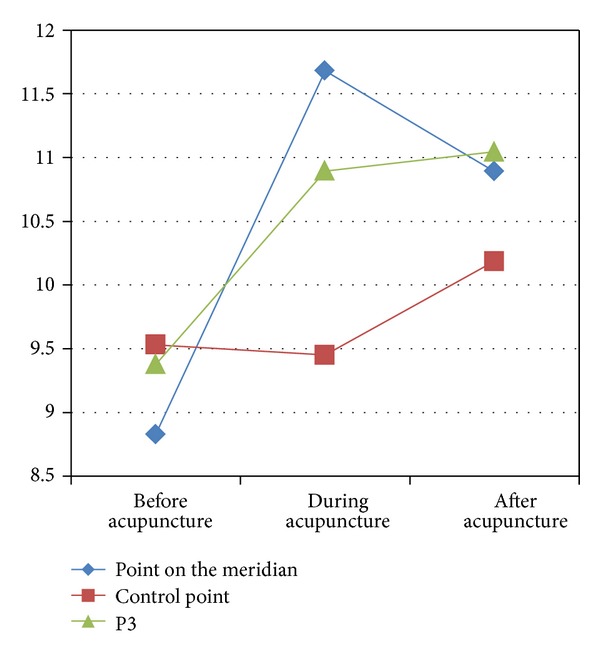
The change of points' TCE caused by Deqi acupuncture.

Similarly, after Deqi acupuncture stimulation, the change of TCE of the point on the meridian and P3 was different from control point. TCE of both points on the meridian and P3 increased during acupuncture and then decreased after acupuncture. On the contrary, TCE of the control point decreased during acupuncture and then increased after acupuncture (see Figure 9). 

**Figure 10:**
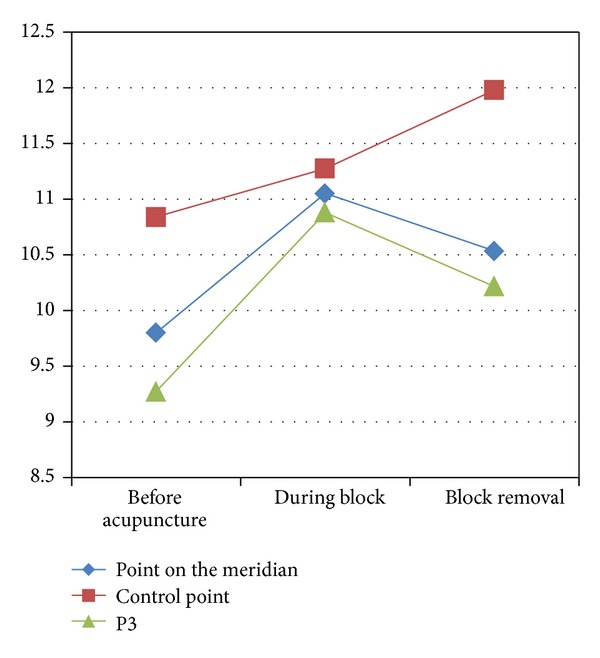
The change of points' TCE caused by acupuncture plus pressure.

When we put pressure on P5, the change of TCE of those points was just like that of Deqi acupuncture (see Figure 10).

**Figure 11:**
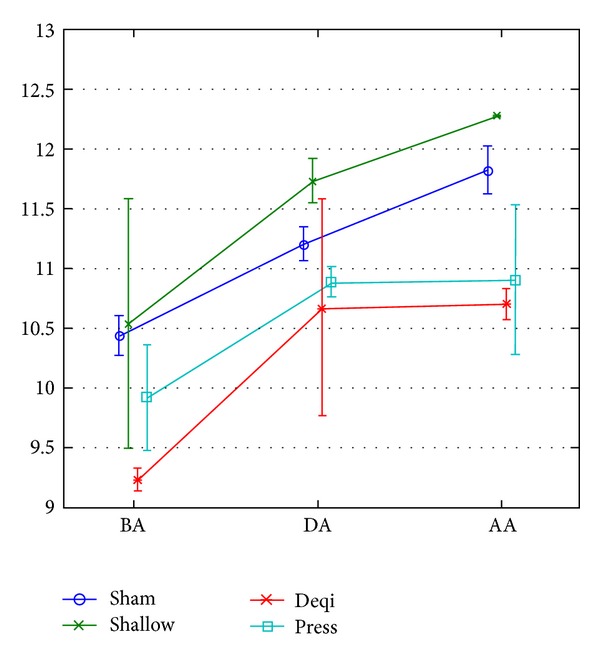
ANOVA for TCE changing before, during, and after acupuncture in 4 types of stimulations.

 In these later 3 conditions, the changes of TCE of control point were similar as if they were not affected by acupuncture stimulation and embodied the specificity of meridian (see Figure 11). 

## 5. Discussion

The neiJing says: “The needle will wander in the channel when you acupuncture in the right Qi point.” [Miraculous Pivot, Lingshu, the 4 chapter]. That means after a doctor stimulates an acupoint, some response will be invoked like Qi arrivals. And only Qi arrival and the effects could be gotten; the faster Qi arrival the faster taking effects. When the Qi is coming, both the manipulator and the patient could feel it. The feeling below the doctor's needle is like heaviness, tense, and fullness, while the patient is feeling aching, distention, tingling, and so on. Some scholars thought that acupuncture might be a special pain stimulus, whose autonomic concomitants could explain its nonanalgesic effects [7]. Zhou's study indicated that different kinds of needling sensations might be associated with different nerve innervations, but it did not explain the relationship between the extension of the sensations and effects [8]. For the sham acupuncture, a study group from Germany thought that sham laser acupuncture could serve as a valid placebo control in laser acupuncture studies. But laser acupuncture is much different from hand acupuncture [9].

In another experiment of the authors, the result showed that the stronger stimulation caused stronger needling sensation [10]. There are more and more similar studies on Deqi recently. For example, Choi did a single-blinded experiment on fifty-three healthy volunteers which received three different forms of acupuncture including superficial needling (0.3 cm) and deep needling (2 cm) to observe the change of the threshold. The result showed that needle rotation and acupuncture sensation play an important role in verifying the effect of acupuncture [11]. 

In this experiment, the authors used questionnaire to investigate the needling sensations after 4 kinds of acupuncture stimulation and found that the traditional distention, aching, and tingling were still the main ones.

TCE was used to measure the performance of the skin energy metabolism of the points. Among 4 stimulations, Deqi acupuncture could get the moderate feeling but with the best curative efficacy. Acupuncture plus pressed P5 caused stronger feeling; therefore, the change of TCE between before and after acupuncture was not obvious. That means the appropriate acupuncture stimulation works best in the clinic, and the needling sensation is not equal to Deqi, positively related to the clinical effects [2].

It is meaningful that the shallow acupuncture could cause the increasing of TCE of both the meridian and the control points. In other words, even the microacupuncture stimulation could improve the whole body energy metabolism, which is nonspecific. on the Contrary, the Deqi acupuncture could improve that significantly. That is why sham acupuncture or acupuncture on “nonacupoint” could get the clinical effects but not as good as Deqi treatment—effective acupuncture stimulus. 

The Qi arrived in Deqi acupuncture; furthermore, the TCE of P3 and Point A which is on the same meridian increased significantly. It showed the existing of specificity of traditional meridian/channel.

## 6. Conclusion

1. Verum acupuncture could cause the stronger needling sensations including distention, aching, numbness, and tingling than sham and shallow acupuncture.
2. The strength of needling sensation caused by Deqi acupuncture is moderate and brought the best curative effects in TCE measurement.
3. Deqi acupuncture could improve the energy metabolism of the points on the corresponding meridian/channel.

## Figures and Tables

**Table 1:** The needling sensation of 4 types acupuncture (number of needling sensations gainer/figures of VAS).

	Heaviness/VAS	Distention/VAS	Aching/VAS	Numbness/VAS	Tingling/VAS	Hot/VAS
Sham		3/1.5		1/2		
Shallow	1	8	5	6	3	
Deqi acupuncture	20/2.8	18/5.33	10/5.4	11/4.05	7/4.21	2/2
Acupuncture + press		14/5.71	9/5.06	13/7.11	3/6.17	
